# Account of a Fistulous Opening in the Stomach

**Published:** 1794

**Authors:** George Burrowes

**Affiliations:** M.R.I.A.


XVII. *Account of a fiſtulous Opening in the Stomach*; *by* George Burrowes, *M. D. M. R. I. A.* Vide *Tranſactions of the Royal Iriſh Academy*, *Vol.* IV.

THE perſon whoſe caſe is here related and who had been an inferior officer in the ſervice of the Eaſt India Company, received, we are told, in a voyage to India, a wound from a blunt-pointed wooden inſtrument in the abdomen, between the cartilage of the eighth rib, on the right ſide, and the umbilicus, penetrating the ſtomach; much inflammation and fever followed the wound, and continued continued a very confiderable time. When the inflammation fubfided an opening remained, through which, when the tent was withdrawn, a fluid of a whitifh colour flowed; the fides, inftead of clofing, turned in, and no union could, by any means, be induced. The man was therefore advifed to keep the opening conftantly plugged up: this he did for the remainder of his life, never withdrawing the plug but to gratify curiofity or to replace it with a new one. The opening was about the third of an inch in diameter. The plug he ufed was generally cotton wick twifted hard.

It was twenty-feven years from the time he received the injury to that at which our author firft faw him. This was about November, 1790, when he was admitted into the Houfe of Induftry at Dublin. He had then attained his fixty-fifth year, and was, to all appearance, we are told, a healthy man, regular in his bowels and in all his fecretions. He had been, it feems, extremely drunken and diffipated, and was, even at that time, frequently intoxicated; yet he never complained of any inconvenience from it, but returned the next day to occupation or debauch with vivacity and with ftrength. He had procured a livelihood for for a few years before Dr. Burrowes met with him by teaching French in Dublin, being too old for his former occupation.—To this account our author adds, that in a voyage ſubſequent to that in which the patient received the wound, he was ſeverely afflicted with ſcurvy, in common with ſeveral others in the ſhip, and in conſequence of that diſeaſe loſt all his teeth. All the alveolar proceſſes were abſorbed, notwithſtanding which he contrived, it ſeems, to break his food, his gums being much hardened, and ate with conſiderable appetite and a good digeſtion.

On removing the plug, after he had taken milk, a part of the milk, we are told, quite pure, eſcaped through the opening; and the patient obſerved that when his ſtomach was empty of meat, and he took the plug out, a whitiſh fluid adhered to it that taſted ſweet. He never felt any pain in the opening, nor inconvenience from any particular food.

After paſſing the winter in the Houſe of Induſtry, he went into the country; but returned at the end of autumn, extremely debilitated, having ſuffered much from hardſhip and intemperance. From this time, it is obſerved, he gradually declined, his appetite continuing tolerably good, but his his bowels weak, till he died, which happened about ſix weeks after his return.

On examining the body after death, the wound was found to penetrate the ſtomach in the centre of the great curvature, and from the adheſions of the liver, colon and integuments, a very conſiderable ſtricture was formed, ſo as to give the ſtomach the appearance of a double bag, with the opening in the middle; the duodenum was enlarged beyond the ſize of the colon, and ſeemed to have in ſome meaſure performed the functions of a ſecond ſtomach. The colon was firmly attached to the lower part of the ſtomach by a ligamentous ſubſtance, that our author thinks, muſt have been formed by the inflammation ſubſequent to the wound. All the other viſcera, he obſerves, were ſound, and perfectly natural both in appearance and ſituation.

Dr. Burrowes concludes the hiſtory of this extraordinary caſe, with expreſſing his regret at his having been prevented from rendering it more ſubſervient to medical purpoſes by the man's ſuddenly departing from the Houſe of Induſtry without his knowledge, and returning to it in ſo debilitated a ſtate; as ſuch an opportunity, his he obſerves, of expoſing aliments to the action of the ſuccus gaſtricus *alone*, of aſcertaining the effects of ſeveral medicines when confined to the ſtomach, and of making experiments on narcotics, he can hardly again expect to meet with.

For accounts of ſimilar caſes Dr. Burrowes refers to *Mem. de l' Acad. Royale de Chirurgie*, Tom. IV. page 124, where, in addition to two ſuch inſtances related by Schenkius, (*Obs. Med.* Lib. III. de *Vuln. Ventric.* Obs. CXXI.) we learn, that M. Foubert preſerved, in his muſeum, the ſtomach of a man who died in the Hotel Dieu at Orleans, which had an opening externally from a wound, and into which the perſon, while alive, uſed frequently to inject different alliments, and digeſted them as well as thoſe taken by the mouth; and that Covillard, in his *Obſervations Iatro-Chirurgiques* (Obs. XLI.) relates the caſe of a ſoldier who received a wound in the upper and lateral part of the epigaſtrium, through which his aliment iſſued. His ſurgeons by means of tents enabled him to retain his food, and by degrees he recovered his health; but the wound of the ſtomach never cloſed, and he was conſtantly obliged to make uſe of a ſilver plug. Covillard made him take out this plug in the preſence of

ſeveral feveral medical men, and they faw about a fpoonful of an imperfectly digefted chylous fubftance iffue from the fiftulous opening; after this he fwallowed a glafs of wine, which was immediately difcharged through the fame paffage. It was obferved that in other refpects the patient had the appearance of a man in perfect health.

